# Identifying the Range of Micro-Events Preceding the Critical Point in the Destruction Process in Traditional and Quasi-Brittle Cement Composites with the Use of a Sound Spectrum

**DOI:** 10.3390/ma14071809

**Published:** 2021-04-06

**Authors:** Dominik Logoń, Janusz Juraszek, Zbynek Keršner, Petr Frantík

**Affiliations:** 1 Faculty of Civil Engineering, Wrocław University of Science and Technology, Wybrzeże Wyspiańskiego 27, 50-370 Wrocław, Poland; 2 Faculty of Materials, Civil and Environmental Engineering, University of Technology and Humanities in Bielsko-Biała, Willowa 2, Building C, 43-309 Bielsko-Biała, Poland; jjuraszek@ath.bielsko.pl; 3 Faculty of Civil Engineering, Brno University of Technology, Veveří 331/95, 602 00 Brno, Czech Republic; kersner.z@fce.vutbr.cz (Z.K.); frantik.p@fce.vutbr.cz (P.F.)

**Keywords:** AE acoustic emission, micro-events, sound spectrum, traditional and quasi-brittle cement composites

## Abstract

This paper presents the possibilities of determining the range of stresses preceding the critical destruction process in cement composites with the use of micro-events identified by means of a sound spectrum. The presented test results refer to the earlier papers in which micro-events (destruction processes) were identified but without determining the stress level of their occurrence. This paper indicates a correlation of 2/3 of the stress level corresponding to the elastic range with the occurrence of micro-events in traditional and quasi-brittle composites. Tests were carried out on beams (with and without reinforcement) subjected to four-point bending. In summary, it is suggested that the conclusions can be extended to other test cases (e.g., compression strength), which should be confirmed by the appropriate tests. The paper also indicates a need for further research to identify micro-events. The correct recognition of micro-events is important for the safety and durability of traditional and quasi-brittle cement composites.

## 1. Introduction

Acoustic Emission (AE) is a well-researched method for testing building materials and structures, which has been used for a very long time in civil engineering [1,2,3,4,5,6]. As a review of the literature shows, papers concentrate on determining the destruction process of materials. AE has been used in monitoring early-age and early hydration acoustic emission of cement paste [7,8]. This method was used to determine the Hooke’s law range and identify the destruction process in cement composites in various cases of loading [9,10,11,12]. AE (events sum AE) has been applied to determine the first crack [13,14,15], micro- and macro-cracks and their propagation in the fracture process in traditional and quasi-brittle cement composites with and without reinforcement [16,17,18,19,20,21,22,23]. Previous tests show the possibility of using AE in various materials, including traditional and high-strength [24,25,26,27] cement composites. The effectiveness of the acoustic emission measurements has been used to monitor structures [28,29,30]. Diagnostics and structure monitoring are still being modified with this method [31,32,33].The majority of papers concerning the destruction process identification are based on the measurement of the AE and AE sum as well as on the spectrograms of the AE signals. Research papers have also demonstrated the possibility of recording the destruction stages with the use of spectrogram images [4].

Various methods of stress determination are still being improved in the process of identification of failure processes in different materials and structures [34,35,36,37,38,39], but the correlation of the stress level with AE in terms of Hooke’s law has not been applied.

Previous research work focused on the correlation between AE and the failure processes of each of the composite components based on the sound spectrum [40,41,42,43,44,45]. It has been found that, for the accurate recognition of composite failure processes, the AE recording should be expanded to include the analysis of each sound separately (as well as a single signal in a very small range of frequencies) and the analysis of the range of sounds corresponding to a given mechanical effect with the use of an acoustic spectrum. It was noticed that the 2D and 3D acoustic spectra should be correlated with the load–deflection curve and with other acoustic effects, which enables the identification of the failure process.

Previous papers [46,47] have focused on the possibility of identifying AE micro-events in the area preceding the occurrence of a critical crack initiating the destruction process in cement composites. It has been indicated that there is a possibility of predicting the occurrence of the f_cr_ (first crack) based on an analysis of the 2D and 3D sound spectra corresponding to the occurring groups of micro-events that precede the end of the load–deflection approximate proportionality area. Tests were conducted on a small-size (40 mm × 40 mm × 160 mm) specimen of a paste with dispersed reinforcement.

This paper indicates the occurrence of micro-events in a wide range of large cement composite beams (paste, mortar and concrete) with traditional and dispersed reinforcement. Before a decision was made on including examples of specimens in this paper, a wide range of composites were analysed and extremely different specimens were selected. The identification of micro-events was carried out by recording the AE on beams with a dimension of 150 mm × 150 mm × 600 mm. Conclusions were drawn based on the analysis of the sound spectrum and the corresponding amplitudes. The repeatability of the results indicates the possibility of considering the conclusions presented in this paper as more generally applicable. The main goal of this paper is the assessment of the level of stress at which micro-events appear that precede the occurrence of the critical crack f_cr_ (LOP—the limit of proportionality) and the flexural strength at bending f_max_ (MOR—the modulus of rupture).

The originality of the presented paper relates to the determination of the stress level (2/3 of the elastic range) for which there occurs the grouping of micro-events in the frequency range and the increase in relative amplitudes of the sound spectrum that precede the destruction process of cement composites. The paper does not identify the types and causes of the micro-events, indicating the need for such research in the future.

## 2. Materials and Methods

The AE tests were carried out on the specimens that were presented in previous papers. The specimens were selected in a manner ensuring that they differ significantly in terms of reinforcement and deformation capacity [43,44,45,46,47,48,49]. Acoustic measurements concentrated on the recording of micro-events. The micro-events were recognized by the space sound spectrum in the range of 0.2–20 kHz.

### 2.1. Materials Used for Tests

Materials for the preparation of the cement composites: c—Portland cement CEM I (class 42.5R) produced by “Górażdże” cement plant in Górażdże (Poland), silica fume (10% of cement mass), fly ash (20% of cement mass), sand of 0–2 mm, superplasticizer (SP) and tap water (w), with the w/b = 0.35 (b = cement + fly ash + silica fume).

Composites:

(1) Mortar without reinforcement—cement:sand (volume) = 1:4.5.
(2) The paste composite was reinforced with dispersed synthetic structural polypropylene fibres (compliance with ASTM C-1116)—specific weight 0.91 kg/dm^3^, flexural strength f_t_ = 620–758 MPa, E = 4.9 GPa, l = 54 mm, equivalent diameter 0.48 mm, l/d = 113 and V_f_ = 6%.
(3) The concrete composite was reinforced with traditional continuous ST500-b reinforcing bars (ArcelorMittal, Warsaw, Poland) with a diameter (d) = 10 mm. Four continuous structural bars in the corners of the beam were placed with stirrups of d = 6 mm, positioned every 150 mm.

### 2.2. Preparation of Specimens for Tests

The ingredients were mixed in the concrete mixer and then used to cast the samples. Beams (150 mm × 150 mm × 600 mm) were cast in slabs and then cured in water at 20 ± 2 °C. After 180 days of ageing, the beams were prepared for the bending tests.

The concrete specimens with traditional reinforcement were not notched. The paste and mortar samples were notched. These beams were turned 90° and cut to the depth of 30 mm. The width of the cut was 3 mm.

### 2.3. Description of the Test Stand

**Figure 1:**
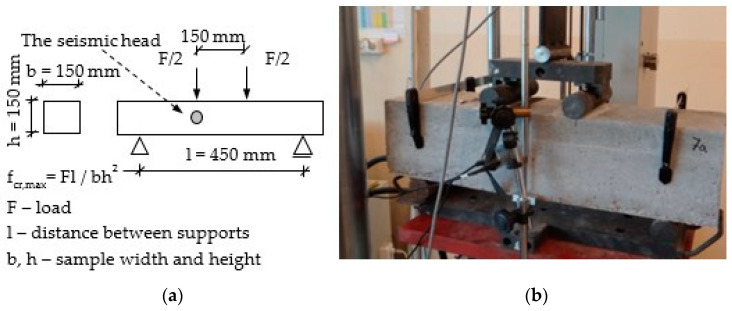
Four-point bending test: the specimen before the test in the test machine: (**a**) diagram of the test [44], (**b**) the specimen before test.

Four-point bending tests were carried out in the testing machine with closed-loop servo control displacement, see Figure 1. The load–deflection curves were obtained according to ASTM C 1018 but the tests were based on the measurement of the continuous and constant displacement of crosshead and the rate was 1 mm/min. The following data were obtained:

- the flexural strength at bending f_max_ (MOR—the modulus of rupture), and the flexural strength at the first crack f_cr_ (LOP—the limit of proportionality);
- the characteristic points on the load–deflection curve, f_x_ (F_x_-load; ε_x-_deflection; W_x_-energy);
- energy (work) as proportional to the area under the load–deflection curve up to the characteristic point.

Acoustic emission effects (AE) were registered and recorded in order to monitor the progress of the cracking process during the monitoring of the load–deflection curve. A seismic head HY919 was used to record the acoustic emission effects in the range from 0.2 to 20 kHz. The sampling rate of the recorded waveforms was 44.1 kHz. The head was placed on the side in the central part of the loaded beams.

The mechanical effects of the composites were correlated with the recorded acoustic spectrum effects. The 3D and 2D sound spectra were measured with the use of the Spectra PLUS-SC program (Pioneer Hill Software LLC, Sequim, WA, USA). The measured AE effects were presented as 3D and 2D acoustic spectra.

**Figure 2:**
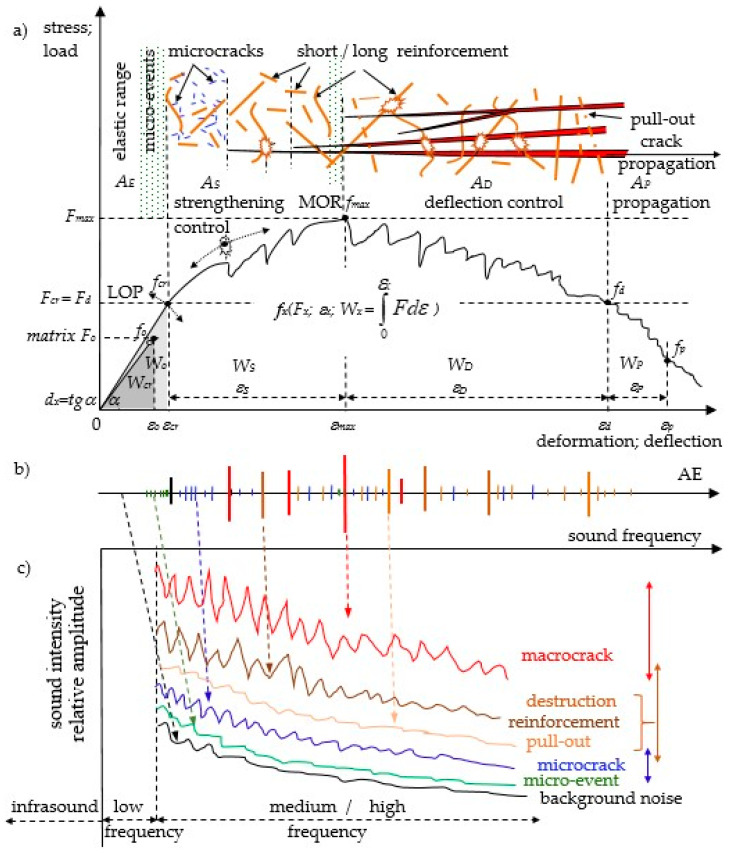
The quasi-brittle composite: (**a**) load–deflection curve; (**b**) AE—acoustic emission effects; (**c**) 2D acoustic spectrum (amplitude depending on sound intensity and frequency).

The load–deflection curves of quasi-brittle cement composites with the corresponding acoustic effects (including sound spectra) are presented. Based on the results included in this paper (and [44]), Figure 2 has been modified by adding micro-events. The spectrum of micro-events was placed directly above the background noise spectrum and below the spectrum corresponding to the micro-crack. The 2D spectra (Figure 2c—amplitude and corresponding frequencies) were determined in relation to an established test duration point correlated with the destruction process, see Figure 2a. The determination of 2D spectra in the time range of the occurrence of the same destruction phenomena increases the range of amplitudes and should not be compared with the spectrum referred to the established test duration point. Infrasound, low- and very high-frequency sounds were not measured; this frequency range is also intended to be recorded in future tests, but it requires heads with a different measurement range.

The ESD reinforcement effect (Eng. elastic range, strengthening control and deflection control) is presented by characteristic points f_x_ (load F_x_; deflection ε_x_; absorbed energy W_x_). The absorbed energy is determined as the area under the load–deflection curve. The following symbols are used: A_E_, W_E_ (the area and absorbed energy corresponding to the elastic range); A_S_, W_S_ (the area and absorbed energy corresponding to the strengthening control); A_D_, W_D_ (the area and absorbed energy corresponding to the deflection control); and A_P_, W_P_ (the area and absorbed energy corresponding to the propagation). Deformation ability was determined as d_x_ = load/deflection.

## 3. Results

**Figure 3:**
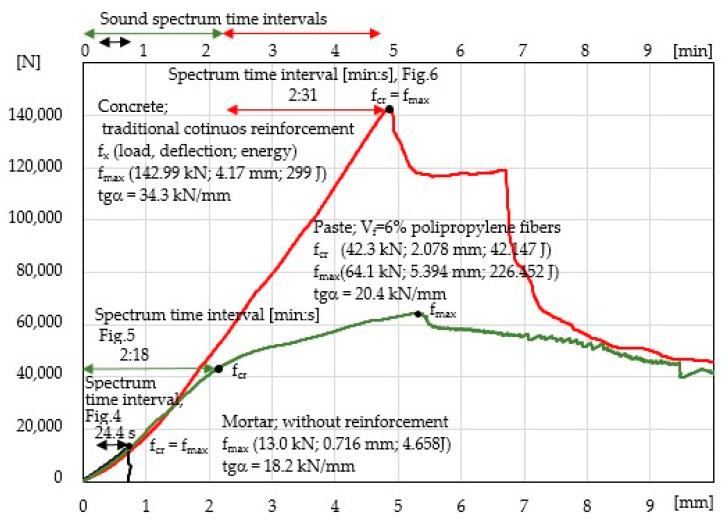
Load–deflection curves of the specimens during the bending test with the analysed sound spectrum time intervals.

Figure 3 presents the examples of the tested specimens in the bending test. A specimen of mortar without reinforcement demonstrates the characteristic catastrophic brittle destruction process after achieving f_cr_ = f_max_. At the critical point f_x_ (load; deflection; energy), the following results were obtained: f_cr_ = f_max_(13.0 kN; 0.71 mm; 4.658 J). The deformation ability is tgα = 18.2 kN/mm.

A cement paste specimen with the maximum possible volume of polypropylene fibres V_f_ = 6% shows a significant improvement in the ability to carry stress in the Hooke’s law range, i.e., f_cr_(42.3 kN; 2.078 mm; 42.147 J), and tgα = 20.4 kN/mm. It is a typical ESD composite with a significant strengthening control range A_s_. The multicracking effects occur in that area. At point f_max_, the following results were obtained: f_max_(64.1 kN; 5.394 mm; 226.452 J), and tgα = 20.4 kN/mm. After that point, further destruction processes based on the sound spectrum in the deflection control area A_D_ and the destruction propagation area A_P_ were not been analysed in this paper.

A concrete specimen with traditional continuous reinforcement demonstrates a strong ability to carry stress in the elastic range A_E_. After critical stress at the characteristic point f_cr_ = f_max_, a macrocrack occurs with f_max_(142.99 kN; 4.17 mm; 299 J) and tgα = 34.3 KN/mm. A significant drop in force is visible after exceeding f_max_ by ca_._ 16%, but a further destruction process is limited by the continuous reinforcement’s ability to carry stress to the deflection of 6.7 mm. Another deformation results in the catastrophic destruction process.

**Figure 4:**
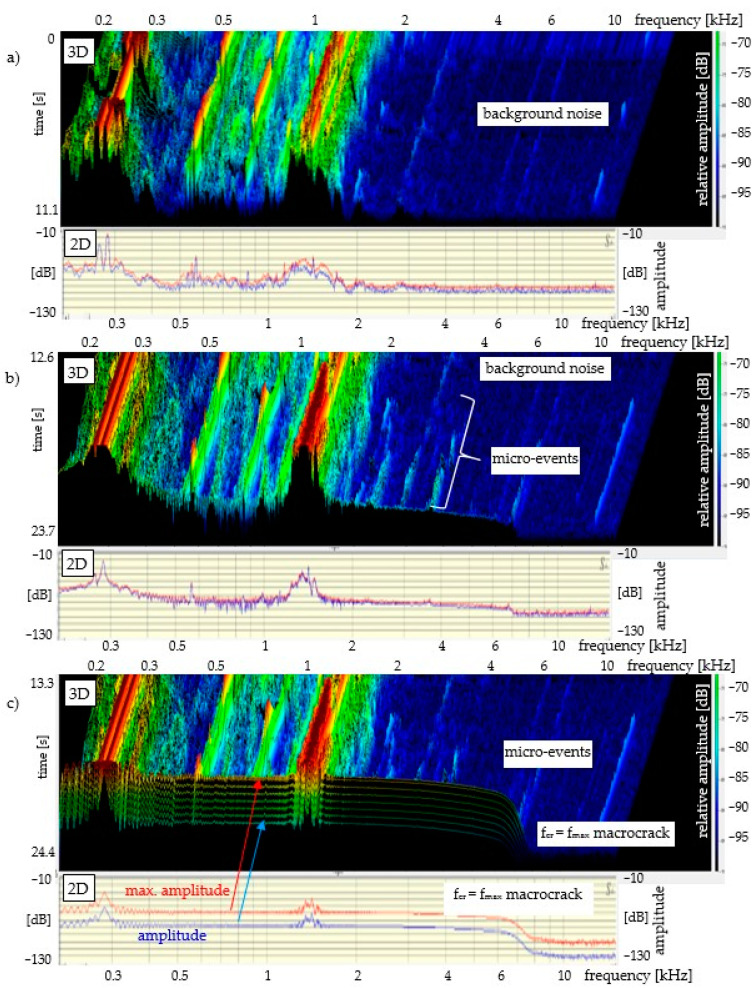
Mortar without reinforcement: bending test—AE; sound spectrum 3D and 2D: (**a**) background noise; (**b**) micro-events; (**c**) micro-events and macrocrack f_cr_ = f_max_.

**Figure 5:**
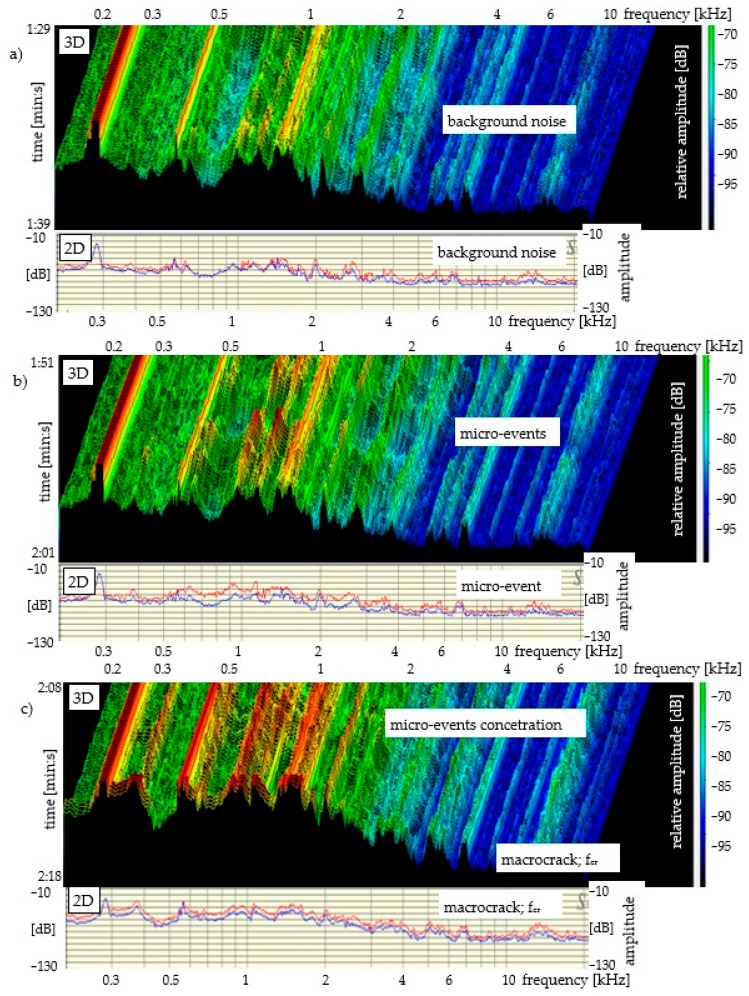
Cement paste with V_f_ = 6% in the bending test and the AE, with 3D and 2D sound spectra: (**a**) background noise; (**b**) micro-events; (**c**) fcr and micro-events concentration.

**Figure 6:**
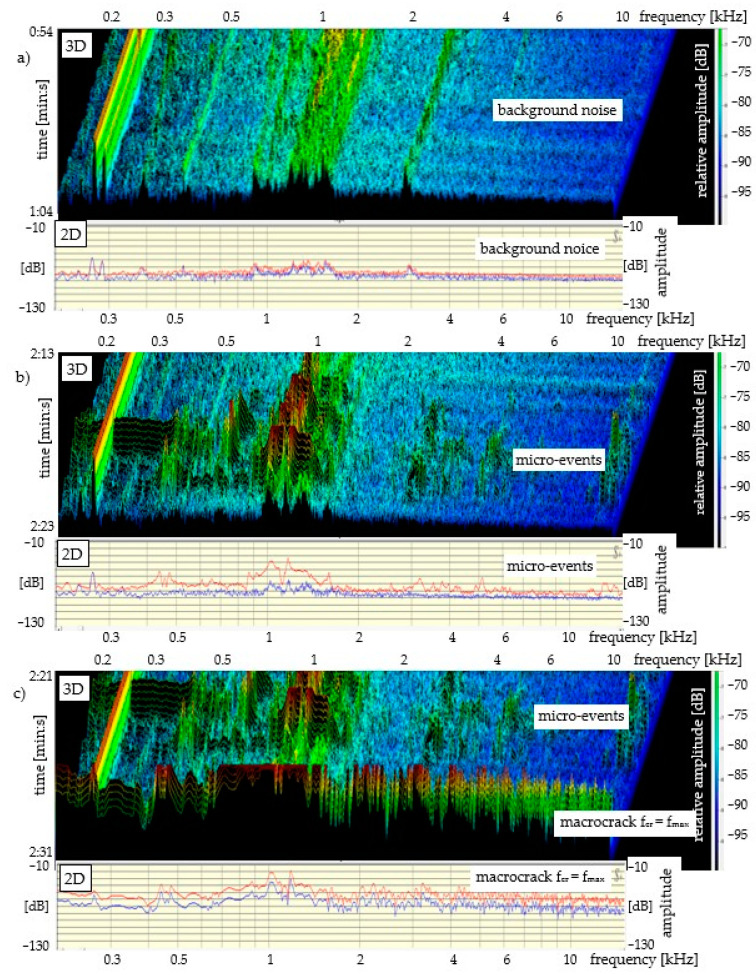
Concrete with traditional continuous reinforcement during the bending test and the AE, with 3D and 2D sound spectra: (**a**) background noise; (**b**) micro-events; (**c**) micro-events and macrocrack f_cr_ = f_max_.

The results of the acoustic emission measurement for all specimens are presented in the same manner (in the same amplitude and frequency range), see Figure 4, Figure 5 and Figure 6. The measurement range of the head limits the AE measurement to the range of 0.2–20 kHz. The lower diagrams present the 3D sound spectrum. Below the 3D spectrum, the 2D spectrum amplitude in the range from −10 to −130 dB was placed. Due to the lack of possibility of presenting all the results from the entire destruction process, only the most characteristic ranges of the sound spectrum are presented, mainly those connected with micro-events preceding f_cr_ and f_max_. The load-deflection test and the AE measurement (mortar and concrete) did not begin at the same time. The time ranges for mortar and concrete precede the occurrence of f_cr_.

Figure 4 presents a 3D and 2D sound spectrum of a mortar specimen without reinforcement (AE measurement during the four-point bending test). The background noise spectrum is presented at the top of Figure 4a. Figure 4b shows micro-events (increase in relative amplitudes before f_cr_). The spectrum corresponding to the critical crack (macrocrack; f_cr_ = f_max_) is shown in Figure 4c. The brittle composite has been destroyed by a macrocrack after reaching the critical point f_cr_ = f_max_. Based on the amplitudes and the frequency range of 0.2–20 kHz of the 2D spectrum, a detailed description can be given of each spectrum corresponding to the elastic range, strengthening control and deflection control. The load-deflection test and the AE measurement (mortar) did not begin at the same time. The duration of the load–deflection test was circa 40 s (Figure 3). The sound spectrum time range was shorter and equalled 24.4 s; this time range preceded the occurrence of f_cr._ The sound spectrum time range was marked on the left-side part of Figure 4c, 3D. Figure 4a presents the 3D sound spectrum in the range from 0 to 11.1 s, which corresponds to the background noise. As is shown in Figure 4b,c, the micro-events begin to concentrate circa 9 s before f_cr_ = f_max_ and then they increase up to f_cr_ = f_max_; this 9-s period of time is within the range between 2/3 of f_cr_ and f_cr_ = f_max_ (load), see Figure 3.

Figure 5 presents the 2D and 3D sound spectra for a beam made of cement paste with dispersed reinforcement V_f_ = 6%. As is shown by the load–deflection correlation in Figure 3, the specimen is a typical ESD composite. The load–deflection test and the AE measurement (cement paste) began at the same time and lasted 138 s (Figure 3). Figure 5a presents the 3D sound spectrum of the background noise whose time range equals 10 s. Micro-events start to appear circa 27 s before f_cr_, then they concentrate circa 8 s before f_cr_ and increase up to f_cr_ = f_max_, see Figure 5b,c; this 27-s period of time (from the moment the micro-events start to appear to f_cr_ ) is within the range of 2/3 of f_cr_ and f_cr_ = f_max_ (load), see Figure 3.

Figure 6 presents the AE results for a concrete specimen reinforced with traditional continuous reinforcement; this specimen is characterised by the largest elastic range. The background noise spectrum is presented in Figure 6a. Figure 6b shows the spectrum of micro-events preceding f_cr_ = f_max_, and Figure 6c presents the spectrum of the macrocrack f_cr_ = f_max_ and the spectrum of micro-events preceding f_cr_. The load–deflection test and the AE measurement (concrete) did not begin at the same time. The duration of the load–deflection test was circa 290 s (Figure 3). The sound spectrum time range was shorter and equalled 91 s; this time range preceded the occurrence of f_cr._ The time was marked on the left-side part of Figure 6c, 3D. Figure 6a presents the 3D sound spectrum of the background noise whose time range equals 10 s. As is shown by Figure 6c, the micro-events begin to concentrate circa 9–10 s before the f_cr_ = f_max_ point; this 9–10-s period of time is within the range of 2/3 of f_cr_ and f_cr_ = f_max_ (load), see Figure 3.

## 4. Discussion

It has been confirmed that the load–deflection curve enables the identification of the proportionality, strengthening, deflection control and crack propagation areas, see Figure 3. The rapid decreases in the ability to carry stress that were recorded on the load–deflection curve indicate the appearance of macrocrack and fibre breaking, see Figure 3, Figure 4, Figure 5 and Figure 6. 

**Figure 7:**
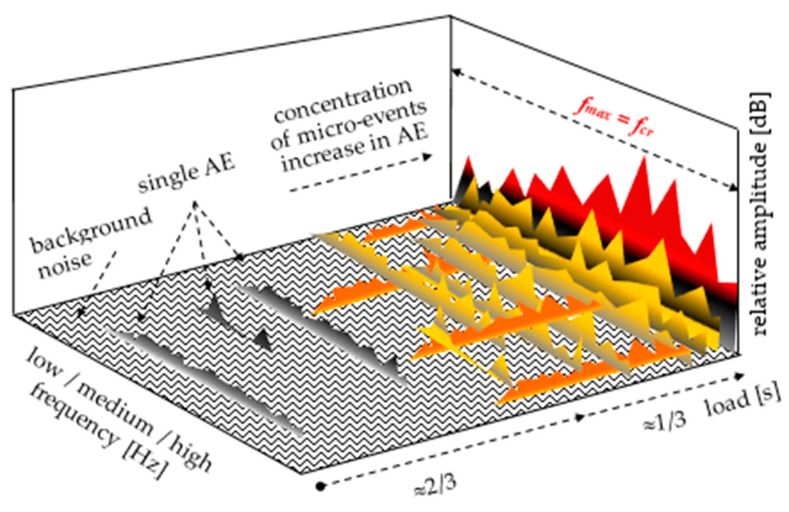
The range of micro-events in cement composites (f_cr_ = f_max_) identified by means of a space sound spectrum.

The recognition of the range of micro-events (using sound spectrum) preceding the critical crack at point f_cr_ = f_max_ is the main focus of research in this paper. In cement composites (with or without reinforcement), which are characterised by a significant decrease in stress after exceeding f_cr_, the recording of micro-events is relatively easy to recognise. The recording of micro-events begins at the point corresponding to circa 2/3 of f_cr_ = f_max_ of the stress in the elastic range, see Figure 4. The concentration of micro-events and the corresponding amplitude range increase as point f_cr_ = f_max_ is being approached. The above observations are illustrated in the form of a diagram in Figure 7. The analysis regarding the range of micro-events occurrence was carried out based on Figure 4, Figure 5 and Figure 6 and the presented data. 

In the case of ESD composites without the decrease in stress after exceeding f_cr_ (e.g., owing to multicracking in the cement paste with V_f_ = 6%, see Figure 3 and Figure 5), micro-events in the elastic range (the same as in composites f_cr_ = f_max_) can be recorded. However, it should be noted that the recognition of the sound spectrum corresponding to f_cr_ as well as the spectra of micro-events preceding f_cr_ is more difficult. This results from the fact that microcracks at point f_cr_ generate smaller acoustic effects than at point f_cr_ = f_max_ (composites characterised by a decrease in stress at the critical point). At the same time, the concentration of events and increase in amplitudes before f_max_ in the tested composite were also recorded. The diversified range of the strengthening control area resulting from the diversified destruction range makes it difficult to estimate the range of occurrence of micro-events before f_max_.

An analysis of the time range of micro-event occurrence recorded in other papers with respect to f_cr_ and f_max_ is similar [46,47]. Micro-events were observed in all other tested specimens, small- and large-size ones, with and without cuts, and with various types of reinforcement. It seems that the conclusions drawn in this respect could be extended to the majority of cement composites.

It should be noted that the speed of crosshead displacement in the process of recognising micro-events in future tests should be significantly reduced. In addition, heads used to record AE should be located in places of applied forces and supports. It has also been noted that the frequency range should be expanded by adding low and high frequency and infrasound spectra, which will probably provide additional information, facilitating the identification of the destruction process.

Micro-events relate to changes at the microstructure level; this destruction process is not visible and requires identification in the future by means of scanning or a microscope. The occurrence of micro-events may be attributed to microcracks connected with the redistribution of stress. They may concentrate in areas subjected to tension or in places where the load is applied. The concentration of stress also occurs at the point of contact between a specimen and the supports on which the beams were placed. The confirmation or refutation of the above suggestions requires additional tests that are planned to be carried out in the near future.

The correct identification of micro-events with the corresponding range of stresses in the elastic range may reduce the level of stresses in the calculations (especially with the cyclical application of dynamic load) in order to increase the durability of structural elements, which so far has not been the focus of interest.

There are lots of variables (location of the sensor, type and geometry of the sensor and sample and background noise) that make it difficult to compare this study with other similar experiments. The identification of micro-events should be expanded for a larger number of specimens with varied geometry, which will serve as the basis for the statistical processing of the results. An attempt to use the presented method conducted under a load–deflection test to monitor building structures based on cement composites is not practical but an effort to record and identify the micro-events (before the occurrence of f_cr_) correlated with the stress level seems to be worth making.

## 5. Conclusions

It has been confirmed that there is a possibility of predicting the occurrence of micro-events in cement composites, which occur at the end of the Hooke’s law range f_cr_ = f_max_.

It has been found that, in cement composites, for which f_cr_ = f_max_, micro-events occur after exceeding 2/3 of the stress elastic range, and the concentration and amplitude of the sound spectrum increase before the critical crack occurs.

Recording micro-events in quasi-brittle cement composites (in which there is no rapid decrease in stress after exceeding critical points f_cr_ and f_max_) is also possible, but the range of occurrence of micro-events and the correlated spectrum amplitudes are smaller, which make it more difficult to locate and recognize them.

The micro-events process is not visible and requires identification in the future by means of scanning or a microscope. This phenomenon may be connected with micro-cracks or regrouping of stresses in the structure of the material or with effects occurring at the place of the applied load, which should be confirmed in future tests. 

The correct identification of micro-events with the corresponding range of stresses in the Hooke’s law area may reduce the level of stresses adopted for the calculation (especially with the cyclical dynamic load), in order to increase the durability of the structural elements.

## Data Availability

No new data were created or analyzed in this study. Data sharing is not applicable to this article.
